# Research on predicting risk factors for re-bleeding in the acute phase of intracerebral hemorrhage using machine learning algorithms

**DOI:** 10.3389/fmed.2026.1855223

**Published:** 2026-06-03

**Authors:** Xiong Deng, JieYao Xia, ZhiJun Liang, Can Luo

**Affiliations:** Department of Neurosurgery, The First Affiliated Hospital of Shaoyang University, Shaoyang, Hunan, China

**Keywords:** GBDT, intracerebral hemorrhage, machine learning, predictive model, re-bleeding, SHAP

## Abstract

**Objective:**

To investigate risk factors for rebleeding in patients during the acute phase of intracerebral hemorrhage, compare the predictive performance of various machine learning models, and develop an optimal predictive model based on SHAP interpretation.

**Methods:**

A retrospective analysis of clinical data from 368 patients with intracerebral hemorrhage was conducted. First, the chi-square test and the Mann–Whitney U test were used to compare the rebleeding group with the non-rebleeding group; Lasso regression was then adopted to screen predictive factors. Based on the six core variables identified, the 10-fold cross-validation performance of nine models, XGBoost, logistic regression, LightGBM, random forest, AdaBoost, decision tree, GBDT, Gaussian Naive Bayes, and k-nearest neighbors, was compared. The most effective GBDT algorithm was used as the foundation, and four additional potential variables were incorporated based on clinical relevance to construct a final GBDT model comprising 10 variables. Bootstrap resampling (1,000 times) was used to calculate the confidence interval for the model’s AUC. Calibration curves and decision curves were employed to evaluate the model’s calibration and clinical utility, while SHAP values were utilized for global and local interpretation. SHAP dependency plots were generated for each feature, and a predictive website was ultimately developed based on this model.

**Results:**

There were statistically significant differences between the two groups in the history of anticoagulant or antiplatelet therapy, hematoma morphology, intraventricular hemorrhage, body temperature, white blood cell count, time from onset to CT scan, hematoma volume, Hematoma Heterogeneity Index (HII), GCS score, lactate, and partial pressure of carbon dioxide (*p* < 0.05). Lasso regression identified six variables: Shape, history of anticoagulant use, time from onset to CT, HII, GCS, and age. In the six-variable model, GBDT achieved the highest AUC (0.967, 95% CI 0.932–1.000) on the validation set. After incorporating DD, intraventricular hemorrhage, history of hypertension, and hematoma volume (V), the final 10-variable GBDT model achieved an AUC of 0.85 on the test set; the mean AUC from bootstrap internal validation was 0.964 (95% CI 0.939–0.982). The calibration curve demonstrated good agreement between the model’s predicted probabilities and actual incidence rates, while the decision curve indicated a net benefit across a wide range of thresholds. The top three variables in the SHAP importance ranking were HII, GCS, and age. The dependency plot showed that a higher HII value, lower GCS score, more irregular hematoma shape, shorter time from onset to CT scan, and larger hematoma volume were all associated with a higher risk of rebleeding.

**Conclusion:**

In this single-center retrospective study, a 10-variable GBDT model accurately predicted early rebleeding risk after acute intracerebral hemorrhage, showing stable Bootstrap performance and improved interpretability via SHAP, which can aid early detection of high-risk patients.

## 1 Introduction

Spontaneous Intracerebral Hemorrhage (SICH) refers to bleeding caused by the rupture of blood vessels within the brain parenchyma due to non-traumatic factors; it is the subtype of stroke with the highest rates of disability and mortality (1). Epidemiological data indicated that intracerebral hemorrhage accounts for approximately 15 to 30% of all stroke types, yet it accounts for more than 40% of stroke-related deaths (2). The incidence of intracerebral hemorrhage in China is approximately 12 ~ 15 per 100,000 people per year, which is significantly higher than in other countries. Patients tend to develop the condition at a relatively young age, placing a heavy burden on society and families (3). Acute-phase rebleeding (Hematoma Expansion (HE)) served as a key event associated with a poor prognosis in patients with intracerebral hemorrhage. Hematoma Expansion (HE) was typically defined as an increase in the original hematoma volume of more than 33% or an increase of more than 6 mL, as shown on a follow-up head CT scan within 24 h of onset (4). Studies indicated that approximately 20 to 40% of patients with intracerebral hemorrhage experience hematoma expansion during the acute phase; patients with hematoma expansion face an increased risk of death by two to three times, and the rate of severe disability rises significantly (5). Although studies have shown that hemostatic agents fail to improve long-term functional outcomes or reduce mortality in patients, and may even increase the risk of systemic thromboembolic complications (6, 7), multiple trials have confirmed that early hemostatic intervention can effectively limit hematoma expansion and improve short-term neurological prognosis (8, 9). Therefore, early identification of high-risk patients for rebleeding and the implementation of targeted interventions (such as intensive blood pressure control, hemostatic therapy, and close monitoring) are crucial for improving patient outcomes. Current clinical practices for predicting hematoma expansion primarily rely on traditional statistical methods. Previous studies have predominantly used multivariate logistic regression to construct risk prediction models, identifying independent risk factors such as baseline hematoma volume, time from onset to imaging, coagulation disorders, and CT findings (e.g., black hole sign, mixed sign, and dot sign) (10, 11). Nevertheless, traditional logistic regression models are limited to capturing nonlinear relationships and complex interactions among variables, and their predictive performance is limited when dealing with high-dimensional clinical data (12). Recent years have witnessed rapid advancements in artificial intelligence technology, and machine learning algorithms have demonstrated significant potential in the field of medical prediction. Ensemble learning algorithms such as gradient-boosted decision trees, extreme gradient boosting, and lightweight gradient boosters can automatically handle nonlinear relationships and interaction effects among variables, and have achieved excellent results in the diagnosis and prognosis prediction of various diseases (13, 14). The “black box” problem of machine learning models, however, limits clinicians’ understanding of and trust in predictive results, thereby hindering their clinical translation and application. SHAP (SHapley Additive Explanations) is a model interpretation method based on Shapley values from game theory. This model quantifies the direction and magnitude of each feature’s contribution to both individual sample predictions and the overall model’s predictions, making the “black-box” models transparent and interpretable (15). This method has been applied in predictive models for cardiovascular diseases, oncology, and neurological disorders (16). This study aimed to collect clinical data, screen reliable variables using Lasso regression, and integrate multiple statistical and machine learning methods to systematically identify risk factors for acute-phase rebleeding in patients with intracerebral hemorrhage. We compared the predictive performance of different models and ultimately constructed an interpretable prediction model based on the gradient boosting decision tree (GBDT) algorithm. Its performance and stability were evaluated via Bootstrap internal validation and SHAP dependence plots, so as to provide decision-making support for early clinical identification of high-risk patients. However, as a single-center retrospective study without external validation, this research has certain limitations.

## 2 Methods

### 2.1 Research subjects

**Figure 1:**
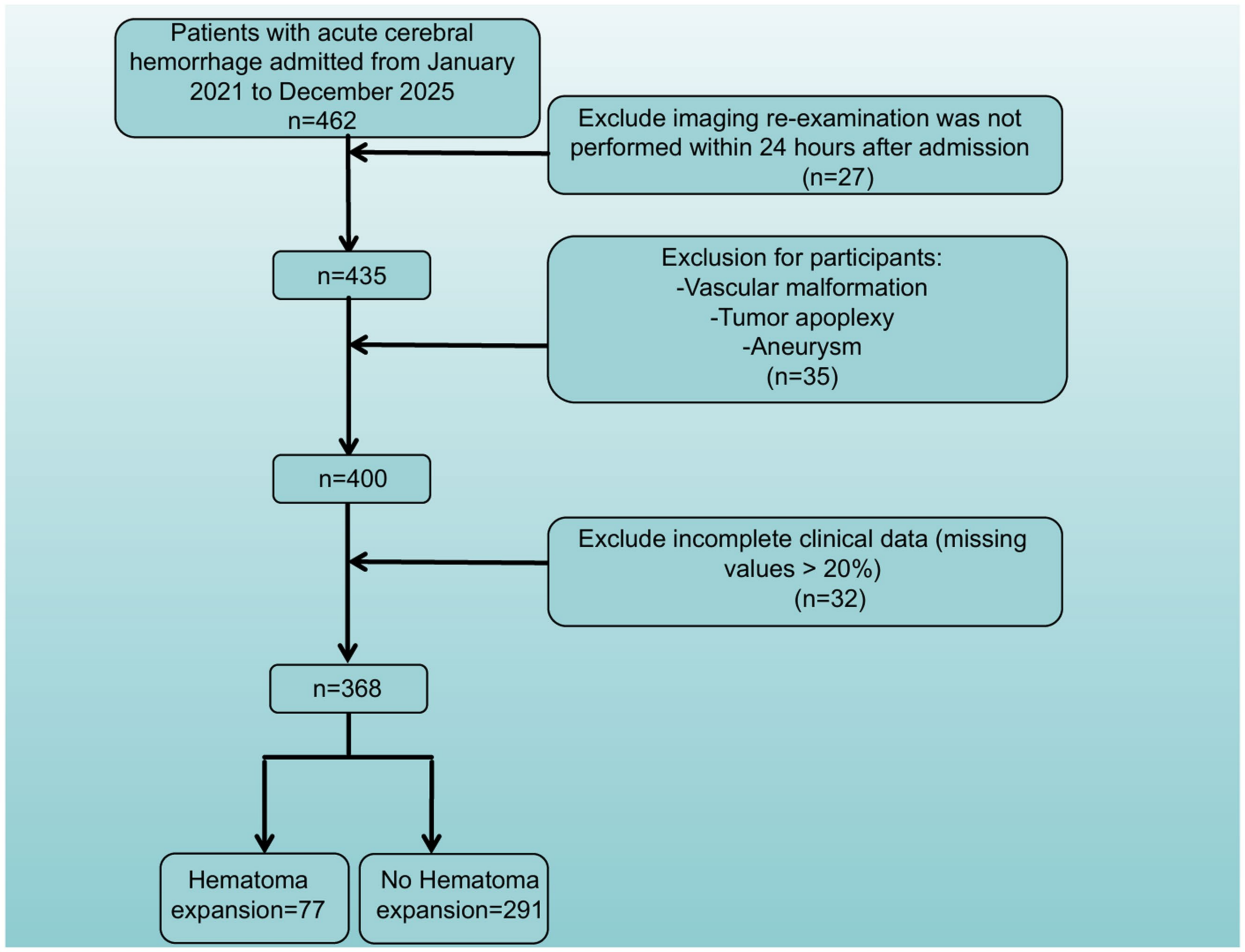
Patient flow chart.

This is a retrospective cohort study that consecutively enrolled 368 patients with acute intracerebral hemorrhage admitted to the Department of Neurosurgery at the First Affiliated Hospital of Shaoyang University between January 2021 and December 2025. Inclusion criteria: (1) age ≥ 18 years; (2) admission within 24 h of symptom onset; (3) diagnosis of spontaneous intracerebral hemorrhage confirmed by an initial cranial CT scan; (4) availability of follow-up CT or MRI imaging data during hospitalization. Exclusion criteria: (1) traumatic intracerebral hemorrhage; (2) secondary intracerebral hemorrhage (e.g., vascular malformations, tumor-related hemorrhage, aneurysms, etc.); (3) failure to undergo imaging follow-up within 24 h of admission; (4) incomplete clinical data (missing values exceeding 20%). Patients were divided into a non-rebleeding group (*n* = 291) and a rebleeding group (*n* = 77) based on whether rebleeding occurred during hospitalization. Rebleeding was defined as: a follow-up head CT scan showing an expansion of the original hematoma volume of ≥33% or an absolute volume increase of ≥6 mL (4). The acute phase of intracerebral hemorrhage is defined as within 24 h after onset (17, 18). This study was approved by the hospital’s Institutional Review Board (Approval No.: K2026-012-01). As this was a retrospective study, informed consent from patients was waived (Figure 1).

### 2.2 Clinical data

The following clinical data were collected from the hospital’s electronic medical record system and imaging system: (1) Demographic characteristics: age, sex, BMI; (2) Medical history: history of hypertension, history of anticoagulant or antiplatelet therapy, underlying conditions (including history of diabetes, coronary heart disease, ischemic stroke, and surgery); (3) Vital signs on admission: temperature (T), pulse (P), respiratory rate (R), blood pressure (BP); (4) Within 1 h of emergency admission laboratory tests: White blood cell count (WBC), hemoglobin (HB), platelet count (PLT), coagulation parameters (TT, APTT, INR, FIB), estimated glomerular filtration rate (eGFR), lactate, blood gas pH, partial pressure of oxygen (PO2), partial pressure of carbon dioxide (PCO2), D-dimer (DD), ALT, AST; (5) Imaging parameters: time from onset to first CT scan, volume of the hematoma at the first scan (V), volume of the hematoma at the second CT scan (V2), hematoma morphology, and intraventricular hemorrhage (IVH). Hematoma volume was calculated using the ABC/2 method, and morphology was classified into three types: subcircular, lobulated, and irregular, as well as mixed and island sign (19); (6) Neurological function scores: Initial Glasgow Coma Scale (GCS) score upon admission. (7) Hematoma Heterogeneity Index (HII) (20), calculated using 3D Slicer, can reflect the internal density heterogeneity and structural instability of the hematoma. Hematoma volume is determined through semi-automated segmentation based on the CT density difference between the hematoma (30 ~ 80 HU) and normal brain parenchyma, with manual correction for partial volume effects to obtain the hematoma surface area (S). The HII formula is: HII=S/π2·33 V/(4π) × 100. (8) Treatment measures: Use of intravenous antihypertensive medications.

### 2.3 Statistical analysis

#### 2.3.1 Univariate analysis

Statistical analysis was performed using SPSS 26.0 and R 4.5.2. Missing values were imputed using a random imputation method. Categorical variables were presented as frequencies and percentages, and comparisons between groups were performed using the chi-square test. Continuous variables were tested for normality using the Shapiro–Wilk test; those that did not follow a normal distribution are presented as the median (interquartile range) [M(P25, P75)], and comparisons between groups were performed using the Mann–Whitney U test. A *p*-value < 0.05 was considered statistically significant.

#### 2.3.2 Lasso regression feature selection

To avoid overfitting and identify important predictive features, Lasso regression was used to perform dimensionality reduction on all candidate variables (21). First, all continuous variables were standardized (Z-score standardization), while categorical variables were encoded. Ten-fold cross-validation was used to determine the optimal penalty parameter *λ*, and variables were selected based on the minimum mean squared error criterion (λmin) and the 1 × standard error criterion (λ1se), respectively (22). The model corresponding to λmin has the lowest cross-validation error but may include a larger number of variables; the model corresponding to λ1se is more parsimonic, with fewer variables but slightly lower predictive performance. Subsequent analyses utilized the core variables selected using λmin.

#### 2.3.3 Pearson correlation analysis

Pearson correlation analysis was used to assess the degree of linear correlation between continuous variables and to identify potential multicollinearity issues. The correlation coefficient (*r*) ranges from −1 to 1; values of |*r*| ≥ 0.5 were considered highly correlated, 0.3 ≤ |*r*| < 0.5 were considered moderately correlated, and |*r*| < 0.3 were considered weakly correlated. The significance level was set at *α* = 0.05. The correlation results were visualized using a heatmap.

### 2.4 Development and comparison of machine learning models

Based on the core variables selected using Lasso, nine classification prediction models were constructed: (1) Extreme Gradient Boosting; (2) Logistic Regression; (3) Lightweight Gradient Boosting Machine; (4) Random Forest; (5) Adaptive Boosting; (6) Decision Trees; (7) Gradient Boosted Decision Trees; (8) Gaussian Naive Bayes; (9) K-Nearest Neighbors. The dataset was randomly split into a training set (70.2%, *n* = 258) and an independent test set (29.8%, *n* = 110), using stratified sampling to ensure consistent sample proportions between the two groups. Ten-fold cross-validation was performed on the training set to tune the parameters, with the AUC of the validation set serving as the primary evaluation metric to compare the performance of each model. An AUC value closer to 1 indicates stronger discriminatory power (23). The model with the highest AUC in the validation set was selected as the base model.

### 2.5 Development and validation of final prediction model

Combining clinical expertise with the results of Pearson correlation analysis, four additional variables clinically considered to be associated with rebleeding (D-dimer, intraventricular hemorrhage, history of hypertension, and hematoma volume V) were incorporated into the core variables selected by Lasso to construct a final GBDT model comprising 10 variables. The model was retrained using the same data splitting and cross-validation strategy. Model performance was evaluated on the independent test set, including AUC, accuracy, sensitivity, specificity, positive predictive value, and negative predictive value. The mean AUC and its 95% confidence interval were calculated using Bootstrap resampling (1,000 times) to assess the model’s stability (24). Calibration curves were used to evaluate the consistency between the model’s predicted probabilities and the actual incidence rates (25). A reliability plot was constructed, and the *p*-value of the Hosmer-Lemeshow test was calculated; a *p*-value > 0.05 indicates good model calibration. The clinical utility of the model was assessed using decision curve analysis (26). The net benefit rate was calculated for different threshold probabilities, decision curves were plotted, and comparisons were made with the “universal intervention” and “no intervention” strategies.

### 2.6 Model interpretation

The SHAP method was used to interpret the final GBDT model (15). The SHAP score, based on the Shapley value theory in game theory, calculated the contribution of each feature to the model’s prediction. Four types of visualizations were adopted to provide global and local explanations: (1) SHAP Summary Plot: Displaying the average absolute SHAP values of each feature relative to the model’s output, reflecting the overall importance of the features; (2) SHAP Dependency Plot: Showing the relationship between changes in individual feature values and their SHAP values, revealing the marginal effects of features on the prediction results and their nonlinear characteristics; (3) SHAP Importance Plot: Displaying the average absolute SHAP values of each feature in ranked order, reflecting the overall magnitude of a feature’s influence on the model’s output, but does not show the direction or distribution of that influence.

### 2.7 Predictive website development

To facilitate clinical implementation, a web-based online risk prediction tool was developed based on the final GBDT prediction model. The 10 clinical indicators selected in the final screening process were used as input features. The tool invokes the loaded GBDT model for real-time inference, calculates the individualized probability of rebleeding, and displays the results. The website interface provides 10 input fields. After clinicians enter the corresponding values and click the “Predict” button, the system instantly outputs the probability of rebleeding during the acute phase of intracerebral hemorrhage, risk stratification, and clinical recommendations.

## 3 Results

### 3.1 Baseline data and univariate analysis

In this study, a total of 368 patients with intracerebral hemorrhage were enrolled, including 77 patients (20.9%) in the rebleeding group and 291 patients (79.1%) in the non-rebleeding group. The results of the univariate analysis are as follows: Comparison of categorical variables revealed statistically significant differences between the two groups in the history of anticoagulant or antiplatelet therapy, hematoma morphology, and intraventricular hemorrhage (*p* < 0.05). In the rebleeding group, the proportion of patients using anticoagulants or antiplatelet agents (18.2% vs. 8.6%), the proportion with irregular hematoma morphology (64.0% vs. 42.3%), and the proportion with concomitant intraventricular hemorrhage (36.4% vs. 19.2%) were all significantly higher than in the non-rebleeding group. After verifying normality using the Shapiro–Wilk test, all continuous variables were found to be skewed or non-normal. Comparisons of continuous variables revealed that white blood cell count (median 9.8 × 109/L vs. 8.2 × 109/L), hematoma volume V (median 25.6 mL vs. 18.3 mL), hematoma heterogeneity index HII (median 137.78 vs. 120.36), and lactate levels (median 2.1 mmol/L vs. 1.7 mmol/L) in the rebleeding group were significantly higher than in the non-rebleeding group (*p* < 0.05); whereas time from onset to CT (median 3.2 h vs. 4.8 h), GCS score (median 11 vs. 14), and PCO2 (median 35.2 mmHg vs. 38.6 mmHg) in the rebleeding group were all significantly lower in the non-rebleeding group (*p* < 0.05). There were no statistically significant differences between the two groups in age, blood pressure, blood glucose, and other parameters (*p* > 0.05) (Tables 1–3).

**Table 1:** Results of cross-tabulation (chi-square) analysis.

Variable	Category	Overview	Non-rebleeding group (*n* = 291)	Rebleeding group (*n* = 77)	χ^2^	*p*
Sex, *n* (%)	Man	122 (33.152)	98 (33.677)	24 (31.169)	0.173	0.678
	Woman	246 (66.848)	193 (66.323)	53 (68.831)		
Hypertension, *n* (%)	No	102 (27.717)	82 (28.179)	20 (25.974)	0.148	0.701
	Yes	266 (72.283)	209 (71.821)	57 (74.026)		
Underlying diseases, *n* (%)	0	65 (17.663)	52 (17.869)	13 (16.883)	5.567	0.350
	1	144 (39.130)	113 (38.832)	31 (40.260)		
	2	106 (28.804)	86 (29.553)	20 (25.974)		
	3	31 (8.424)	25 (8.591)	6 (7.792)		
	4	18 (4.891)	11 (3.780)	7 (9.091)		
	5	4 (1.087)	4 (1.375)	0 (0.000)		
Anticoagulant or antiplatelet, *n* (%)	No	333 (90.489)	269 (92.440)	64 (83.117)	6.149	**0.013**
	Yes	35 (9.511)	22 (7.560)	13 (16.883)		
Shape, *n* (%)	1	214 (58.152)	199 (68.385)	15 (19.481)	84.533	**<0.001**
	2	108 (29.348)	76 (26.117)	32 (41.558)		
	3	46 (12.500)	16 (5.498)	30 (38.961)		
Intraventricular extension, *n* (%)	No	295 (80.163)	250 (85.911)	45 (58.442)	28.892	**<0.001**
	Yes	73 (19.837)	41 (14.089)	32 (41.558)		
BP, *n* (%)	0	86 (23.370)	70 (24.055)	16 (20.779)	0.595	0.898
	I	124 (33.696)	98 (33.677)	26 (33.766)		
	II	81 (22.011)	62 (21.306)	19 (24.675)		
	III	77 (20.924)	61 (20.962)	16 (20.779)		
IV antihypertensive agents, *n* (%)	No	152 (41.304)	126 (43.299)	26 (33.766)	2.282	0.131
	Yes	216 (58.696)	165 (56.701)	51 (66.234)		

BP, blood pressure; According to the hypertension grading, no hypertension was assigned a score of 0; IV, intravenous; Underlying diseases include a history of diabetes, coronary heart disease, cerebral infarction, and surgery, with a score of 0 if none is present and an increase of 1 for each condition present; Hematoma shape was classified as Shape = 1 (regular: round/oval), Shape = 2 (irregular: lobulated/irregular), and Shape = 3 (mixed density/island sign). Bold type indicates *P* < 0.05.

**Table 2:** Shapiro–Wilk test.

Variable	Skewness	Kurtosis	SE	W	*p*
Age	−0.414	0.320	0.662	0.983	0.000
T	3.030	17.773	0.022	0.749	0.000
P	0.716	1.573	0.842	0.972	0.000
R	1.455	4.777	0.165	0.830	0.000
eGFR	−0.675	0.346	1.709	0.931	0.000
WBC	1.049	1.395	0.208	0.936	0.000
HB	−0.857	3.439	1.223	0.953	0.000
PLT	0.529	1.423	3.676	0.977	0.000
Time from on set to CT	0.790	−0.785	0.366	0.859	0.000
V2	1.876	4.057	1.570	0.789	0.000
V	1.992	5.034	1.262	0.792	0.000
HII	0.710	−0.765	0.412	0.892	0.000
GCS	−0.837	−0.649	0.188	0.847	0.000
ALT	12.726	204.080	1.775	0.316	0.000
TT	0.428	3.539	0.074	0.956	0.000
AST	4.250	23.479	1.211	0.578	0.000
DD	9.843	132.546	0.204	0.314	0.000
INR	5.475	42.852	0.008	0.582	0.000
APTT	6.956	91.449	0.228	0.646	0.000
FIB	1.633	4.867	0.058	0.885	0.000
PH	−0.871	6.828	0.003	0.926	0.000
Lactate	4.253	28.666	0.077	0.659	0.000
PO2	1.481	7.491	1.780	0.922	0.000
PCO2	1.763	17.462	0.364	0.873	0.000
BMI	0.542	5.946	0.158	0.926	0.000

T, Temperature; P, Pulse; R, Respiration; eGFR, estimated glomerular filtration rate; WBC, white blood cell; Hb, hemoglobin; PLT, platelet; V, hematoma volume; V2, secondary hematoma volume; HII, Hemorrhage Heterogeneity Index; GCS, Glasgow Coma Scale; ALT, alanine aminotransferase; AST, aspartate aminotransferase; TT, thrombin time; DD, D-dimer; INR, international normalized ratio; APTT, activated partial thromboplastin time; FIB, fibrinogen; pH, potential of hydrogen; Lactate, blood lactate; PO₂, partial pressure of oxygen; PCO₂, partial pressure of carbon dioxide; BMI, body mass index.

**Table 3:** Analysis of variance.

Variable	Overview	Non-increased hemorrhage (*n* = 291)	ncreased hemorrhage (*n* = 77)	U	*p*
Age (year), median [IQR]	64.000 [54.000,72.000]	64.000 [55.000,72.000]	63.000 [52.000,74.000]	0.199	0.842
T (°C), median [IQR]	36.800 [36.500,36.900]	36.700 [36.500,36.900]	36.800 [36.600,36.900]	−2.311	**0.019**
P (beats/min), median [IQR]	80.000 [70.000,90.000]	80.000 [70.000,90.000]	80.000 [70.000,91.000]	−0.565	0.572
R (beats/min), median [IQR]	20.000 [18.000,20.000]	20.000 [19.000,20.000]	20.000 [18.000,21.000]	1.061	0.262
eGFR (mL·min^−1^·1.73 m^−2^), median [IQR]	90.000 [66.500,104.100]	90.000 [67.300,102.300]	90.700 [64.800,108.000]	−1.028	0.304
WBC (×10^9^/L), median [IQR]	8.790 [6.830,11.970]	8.560 [6.670,11.780]	9.750 [7.540,12.920]	−2.451	**0.014**
HB (g/L),median [IQR]	137.000 [122.000,148.000]	137.000 [122.000,147.000]	137.000 [121.000,152.000]	−0.320	0.749
PLT (×10^9^/L),median [IQR]	195.000 [164.000,237.000]	197.000 [166.000,239.000]	185.000 [149.000,235.000]	1.423	0.155
Time from on set to CT (h),median [IQR]	6.000 [4.000,14.000]	7.000 [4.000,16.000]	5.000 [3.000,8.000]	3.189	**0.001**
V (mL),median [IQR]	15.346 [6.300,29.500]	12.300 [5.200,23.900]	26.700 [17.800,47.600]	−6.498	**<0.001**
V2 (mL),median [IQR]	16.200 [6.500,39.200]	12.900 [5.300,23.600]	53.600 [27.800,70.300]	−8.990	**<0.001**
HII,median [IQR]	121.450 [118.340,129.120]	120.361 [117.450,124.670]	137.780 [129.340,138.780]	−10.767	**<0.001**
GCS, median [IQR]	13.000 [9.000,14.000]	13.000 [10.000,14.000]	7.160 [6.000,11.000]	6.884	**<0.001**
ALT (U/L), median [IQR]	18.900 [13.100,28.300]	18.300 [13.000,26.700]	20.800 [13.800,34.900]	−1.621	0.105
AST (U/L), median [IQR]	23.200 [19.100,32.400]	23.000 [19.000,31.900]	25.400 [20.600,34.500]	−1.735	0.083
DD (mg/L FEU), median [IQR]	0.470 [0.220,1.180]	0.470 [0.220,1.090]	0.470 [0.210,1.250]	−0.473	0.637
INR (ratio), median [IQR]	0.970 [0.920,1.030]	0.970 [0.920,1.030]	0.980 [0.930,1.030]	−0.386	0.700
TT (s), median [IQR]	18.200 [17.300,18.900]	18.200 [17.300,18.900]	18.300 [17.500,19.200]	−0.960	0.337
FIB (g/L), median [IQR]	3.010 [2.500,3.520]	3.010 [2.580,3.530]	2.850 [2.370,3.400]	1.428	0.154
APTT (s), median [IQR]	25.100 [23.300,27.100]	25.100 [23.300,27.200]	24.900 [23.100,26.200]	1.316	0.188
PH, median [IQR]	7.400 [7.370,7.430]	7.400 [7.370,7.420]	7.400 [7.380,7.430]	−0.430	0.667
PO2 (mmHg), median [IQR]	112.000 [88.000,129.000]	112.000 [88.000,125.000]	114.612 [89.000,141.000]	−1.113	0.266
Lactate (mmol/L), median [IQR]	1.600 [1.162,2.200]	1.560 [1.100,2.013]	1.700 [1.300,2.600]	−2.326	**0.020**
PCO2 (mmHg),median [IQR]	38.200 [34.600,40.700]	38.541 [35.200,40.800]	36.938 [32.300,40.700]	2.341	**0.019**
BMI (kg/m^2^), median [IQR]	23.878 [22.382,24.949]	23.805 [22.278,24.859]	24.159 [22.794,25.360]	−1.543	0.123

T, Temperature; P, Pulse; R, Respiration; eGFR, estimated glomerular filtration rate; WBC, white blood cell; Hb, hemoglobin; PLT, platelet; V, hematoma volume; V2, secondary hematoma volume; HII, Hematoma Heterogeneity Index; GCS, Glasgow Coma Scale; ALT, alanine aminotransferase; AST, aspartate aminotransferase; TT, thrombin time; DD, D-dimer; INR, international normalized ratio; APTT, activated partial thromboplastin time; FIB, fibrinogen; pH, potential of hydrogen; Lactate, blood lactate; PO₂, partial pressure of oxygen; PCO₂, partial pressure of carbon dioxide; BMI, body mass index. Bold type indicates *p* < 0.05.

### 3.2 Pearson correlation analysis

**Figure 2:**
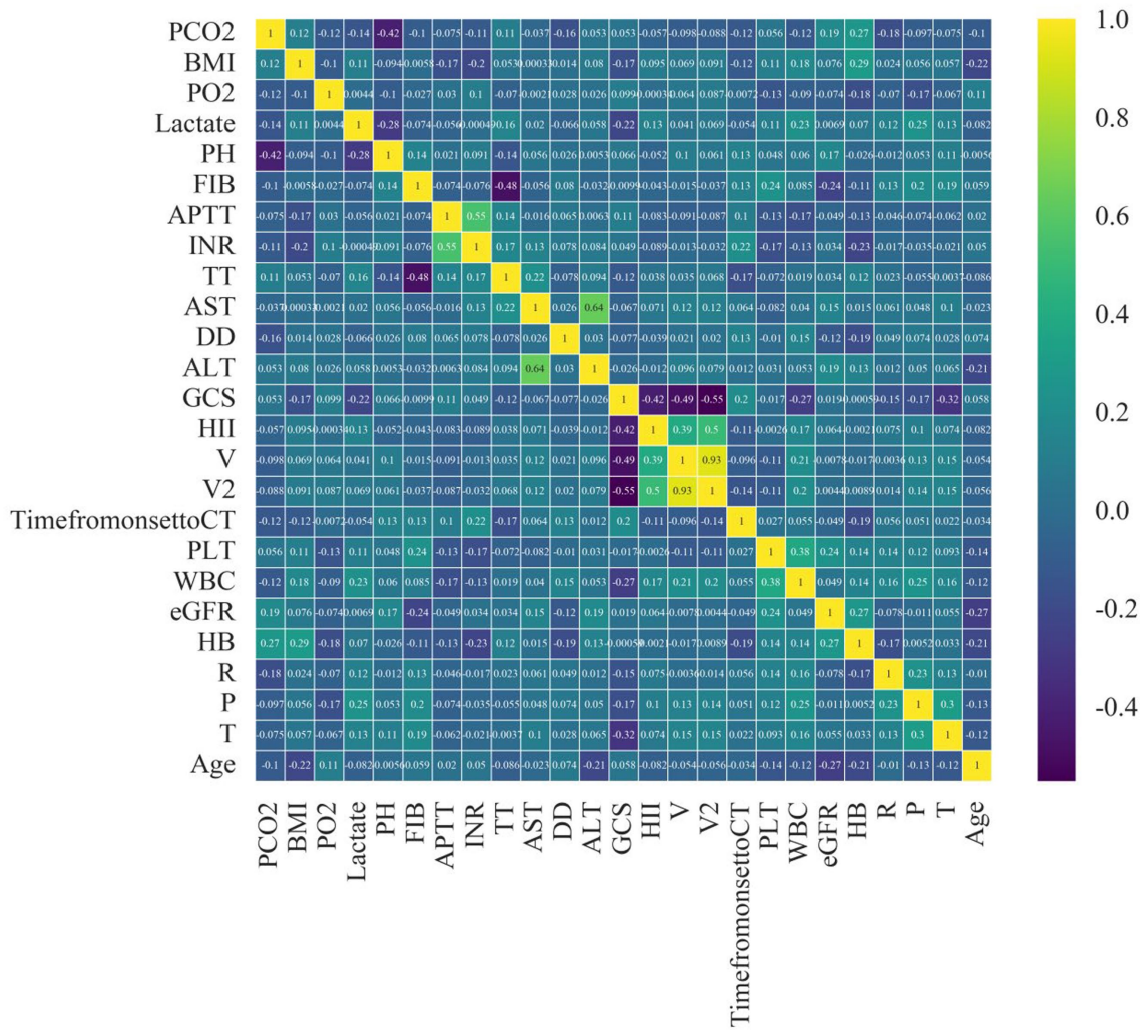
Spearmanrank correlation heatmap of variables.

Pearson correlation analysis revealed significant correlations between some continuous variables (*p* < 0.05). Variables with a high correlation (|*r*| ≥ 0.5) included hematoma volume (V) and the square of hematoma volume (V2): *r* = 0.932, *p* < 0.001. These two variables showed a strong positive correlation, consistent with mathematical relationships. GCS score and V2: *r* = −0.553, *p* < 0.001, suggesting that the larger the hematoma volume, the more severe the patient’s level of impaired consciousness. APTT and INR: *r* = 0.548, *p* < 0.001; as both are indicators of coagulation function, they show a high degree of consistency. AST and ALT: *r* = 0.642, *p* < 0.001, indicating a strong correlation between these two markers of liver function. HII and V2: *r* = 0.501, *p* < 0.001, suggesting that patients with larger hematoma volumes also have higher HII values in their imaging characteristics. Furthermore, GCS showed a moderate negative correlation with HII (*r* = −0.425, *p* < 0.001) and a moderate negative correlation with V (*r* = −0.487, *p* < 0.001), while HII showed a moderate positive correlation with V (*r* = 0.391, *p* < 0.001) (Figure 2).

### 3.3 Lasso regression feature selection

**Figure 3:**
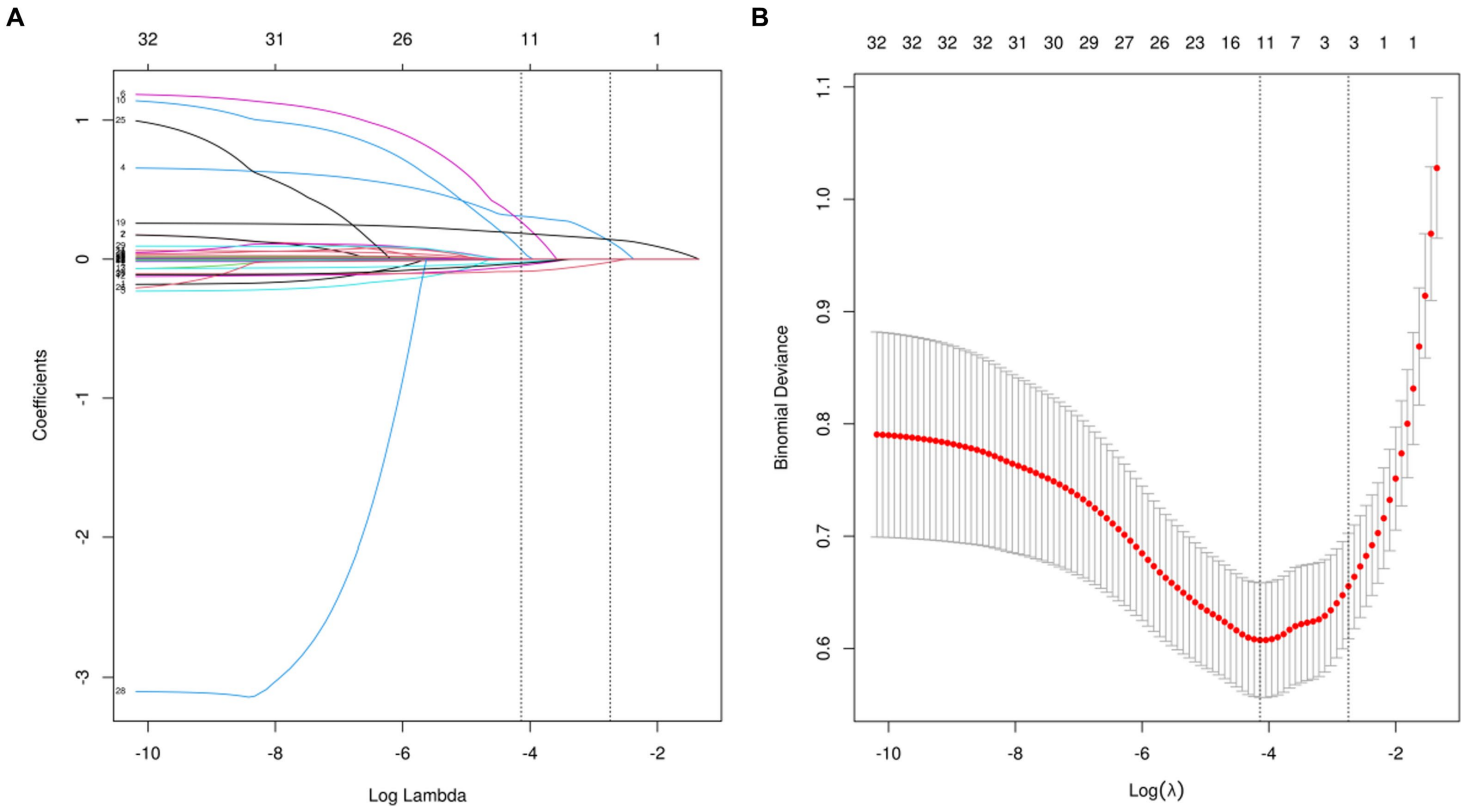
**(A)** Coefficient profile plot of Lasso regression. **(B)** Cross-validation curve plot of Lasso regression.

The results of 10-fold cross-validation for Lasso regression (Figures 3A,B) showed that when *λ* was set to its minimum value of 0.015 (λmin), six variables with non-zero coefficients were identified, in order: hematoma shape (Shape), history of anticoagulant or antiplatelet therapy, time from onset to CT scan, HII, GCS score, and age. When λ was set to 1 times the standard error (0.071, λ1se), 3 core variables were retained: Shape, HII, and GCS. Considering clinical utility, subsequent multi-model comparisons were based on the 6 variables selected using λmin.

### 3.4 Comparison of multiple machine learning models

**Figure 4:**
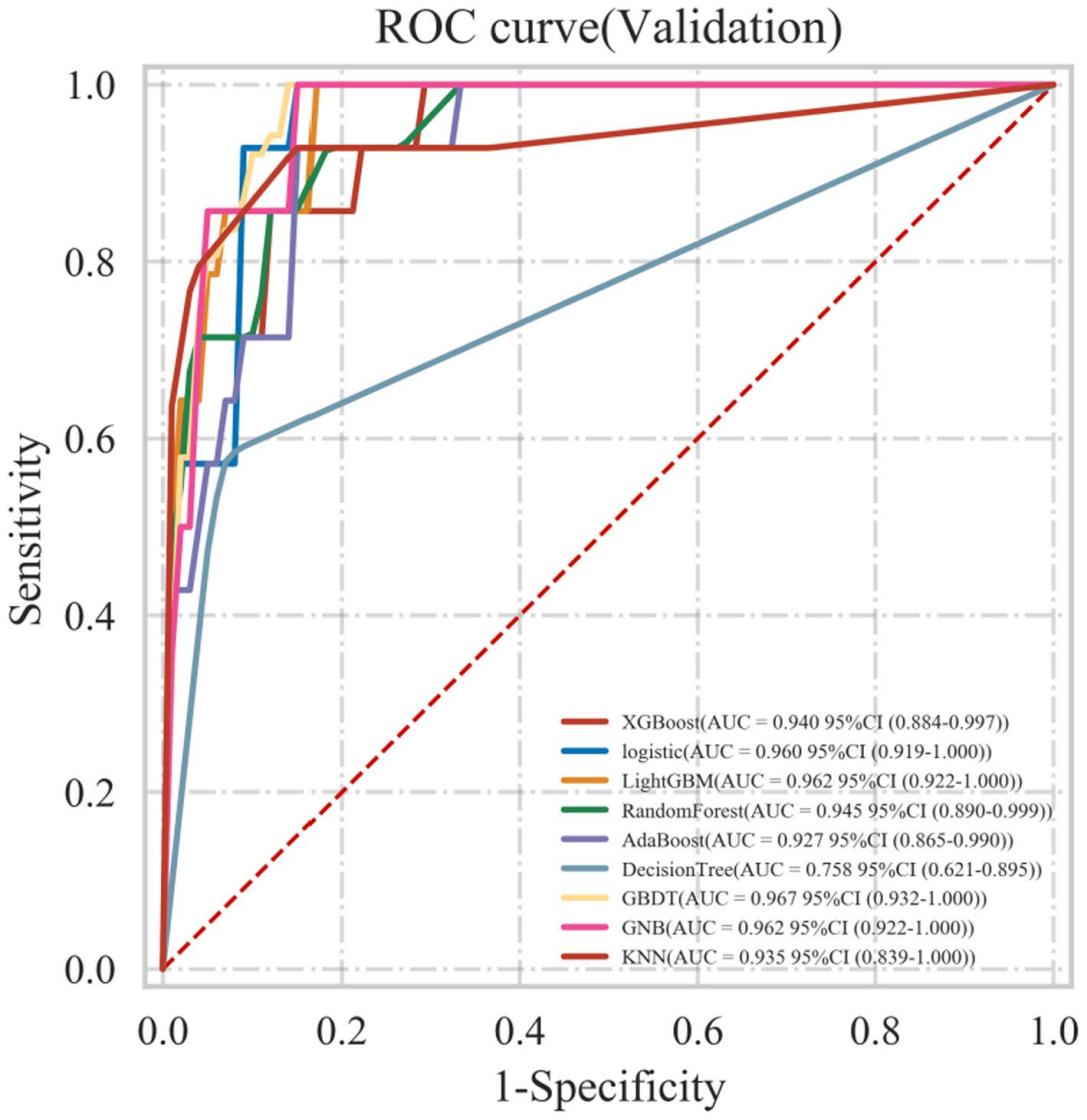
ROC curve for machine learning training.

Figure 1 showed a comparison of the performance of nine machine learning models on a 10-fold cross-validation validation set based on six core variables. The GBDT model performed best, achieving an AUC of 0.967 (95% CI 0.932–1.000) on the validation set, with an accuracy of 0.907, a sensitivity of 0.857, and a specificity of 0.918. The XGBoost model achieved an AUC of 1.000 on the training set but 0.940 on the validation set, indicating a risk of overfitting. The logistic regression model achieved an AUC of 0.892 on the validation set, demonstrating relatively stable performance but lower efficacy. Detailed performance metrics for each model were presented in Table 4 and Figure 4.

**Table 4:** Diagnostic efficacy of different machine learning classification models.

Model	AUC (95%CI)	Cutoff (95%CI)	Accuracy (95%CI)	Sensitivity (95%CI)	Specificity (95%CI)	Positive predictive value (95%CI)	Negative predictive value (95%CI)	F1 (95%CI)	Kappa (95%CI)
XGBoost	0.940 (0.884–0.997)	0.534 (0.534–0.534)	0.919 (0.919–0.919)	0.714 (0.714–0.714)	0.967 (0.967–0.967)	0.833 (0.833–0.833)	0.935 (0.935–0.935)	0.769 (0.769–0.769)	0.72 (0.720–0.720)
logistic	0.960 (0.919–1.000)	0.354 (0.354–0.354)	0.919 (0.919–0.919)	0.929 (0.929–0.929)	0.917 (0.917–0.917)	0.722 (0.722–0.722)	0.982 (0.982–0.982)	0.813 (0.813–0.813)	0.762 (0.762–0.762)
LightGBM	0.962 (0.922–1.000)	0.712 (0.712–0.712)	0.919 (0.919–0.919)	0.643 (0.643–0.643)	0.983 (0.983–0.983)	0.9 (0.900–0.900)	0.922 (0.922–0.922)	0.75 (0.750–0.750)	0.703 (0.703–0.703)
RandomForest	0.945 (0.890–0.999)	0.5 (0.500–0.500)	0.865 (0.865–0.865)	0.714 (0.714–0.714)	0.9 (0.900–0.900)	0.625 (0.625–0.625)	0.931 (0.931–0.931)	0.667 (0.667–0.667)	0.582 (0.582–0.582)
AdaBoost	0.927 (0.865–0.990)	0.491 (0.491–0.491)	0.851 (0.851–0.851)	0.929 (0.929–0.929)	0.833 (0.833–0.833)	0.565 (0.565–0.565)	0.98 (0.980–0.980)	0.703 (0.703–0.703)	0.611 (0.611–0.611)
DecisionTree	0.758 (0.621–0.895)	1.0 (1.000–1.000)	0.869 (0.860–0.878)	0.579 (0.546–0.611)	0.937 (0.930–0.943)	0.681 (0.653–0.709)	0.905 (0.898–0.912)	0.625 (0.597–0.653)	0.546 (0.514–0.579)
GBDT	0.967 (0.932–1.000)	0.312 (0.302–0.322)	0.907 (0.904–0.909)	0.857 (0.857–0.857)	0.918 (0.915–0.922)	0.71 (0.702–0.719)	0.965 (0.965–0.965)	0.777 (0.772–0.782)	0.719 (0.712–0.725)
GNB	0.962 (0.922–1.000)	0.619 (0.619–0.619)	0.905 (0.905–0.905)	0.857 (0.857–0.857)	0.917 (0.917–0.917)	0.706 (0.706–0.706)	0.965 (0.965–0.965)	0.774 (0.774–0.774)	0.715 (0.715–0.715)
KNN	0.935 (0.839–1.000)	0.4 (0.400–0.400)	0.865 (0.865–0.865)	0.929 (0.929–0.929)	0.85 (0.850–0.850)	0.591 (0.591–0.591)	0.981 (0.981–0.981)	0.722 (0.722–0.722)	0.639 (0.639–0.639)

### 3.5 GBDT model performance

**Figure 5:**
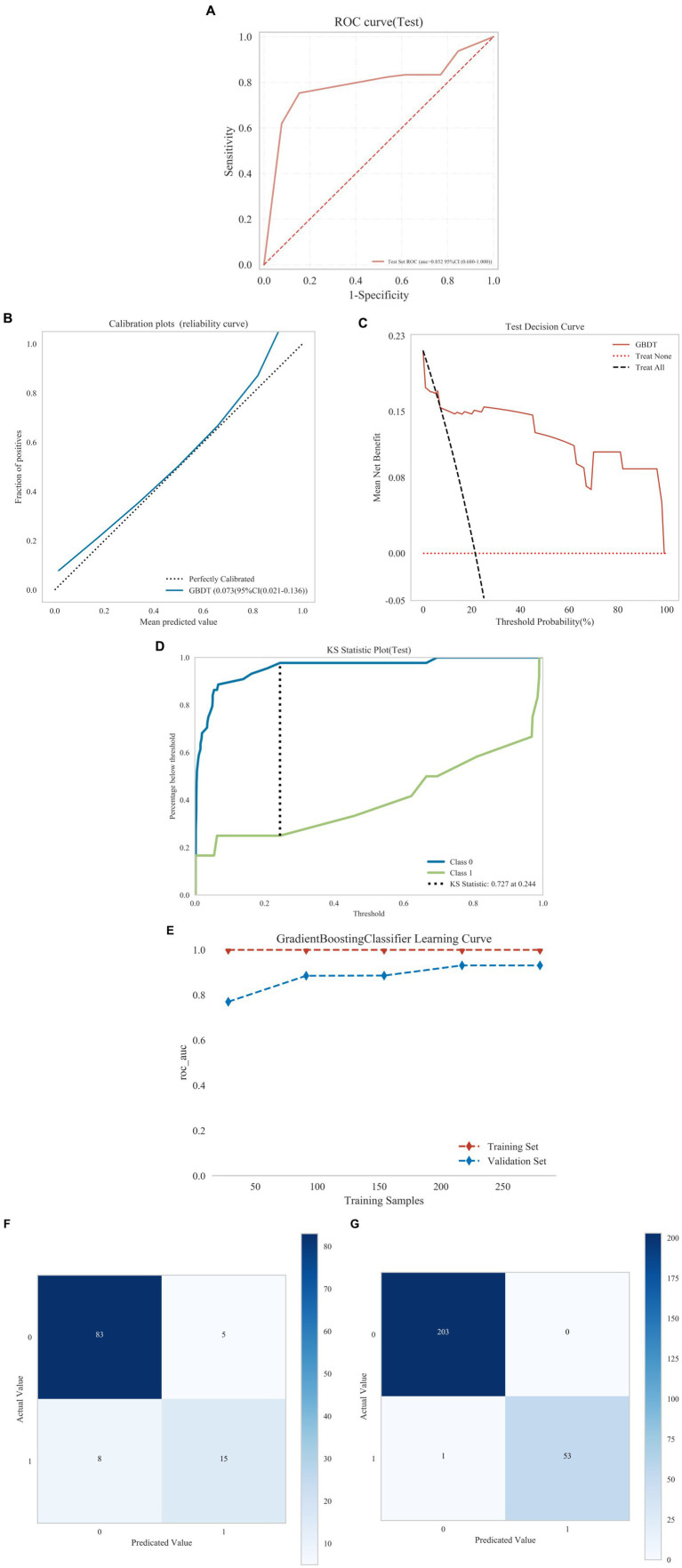
**(A)** ROC curve of 10-variable GBDT model. **(B)** Calibration curve of the 10-variable GBDT model. **(C)** Decision curve analysis of the 10-variable GBDT model. **(D)** Kolmogorov–Smirnov (KS) curve of the 10-variable GBDT model (0: no hematoma expansion; 1: hematoma expansion). **(E)** Learning curve of the 10-variable GBDT model. **(F)** Confusion matrix of the 10-variable GBDT model in the test set. **(G)** Confusion matrix of the 10-variable GBDT model in the training set.

**Figure 6:**
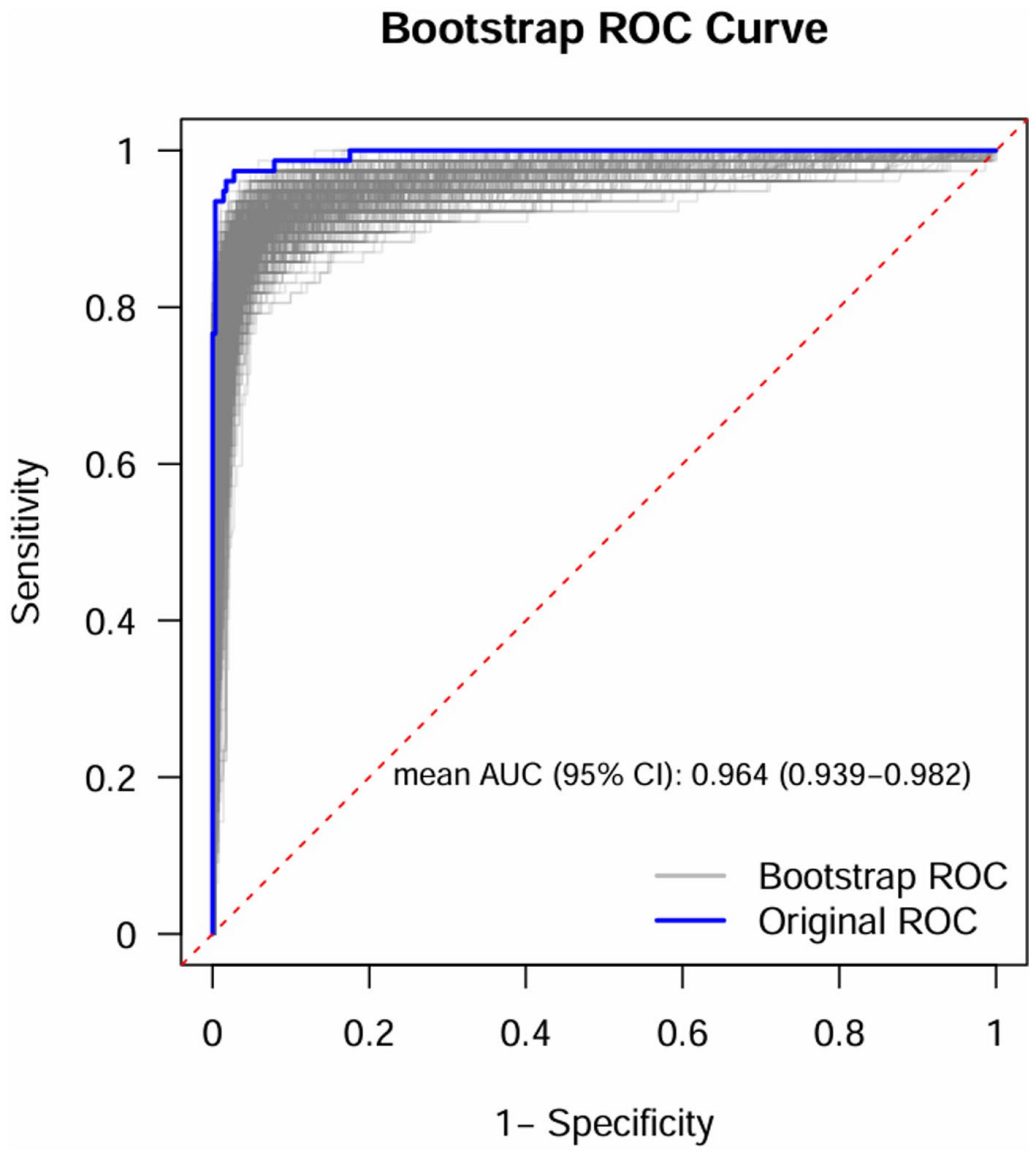
Bootstrap AUC curve of the 10-variable GBDT model.

After incorporating four additional potential variables, D-dimer (DD), intraventricular hemorrhage (IVH), history of hypertension, and hematoma volume (V), into the original six-variable model, the final 10-variable GBDT model achieved the following performance metrics on the independent test set: AUC = 0.85, accuracy = 0.8829, sensitivity = 0.652, and specificity = 0.938. The confusion matrix for the test set showed: 83 correctly classified negative cases and 15 correctly classified positive cases; 5 false-positive misclassifications and 8 false-negative misclassifications (Figures 5A–G). The results of bootstrap internal validation (1,000 resamples) showed a mean AUC of 0.964 with a 95% confidence interval of 0.939 ~ 0.982, indicating that the model possesses good stability and generalization ability. The calibration curve showed that the model’s predicted probabilities were generally consistent with the observed frequencies; the Hosmer-Lemeshow test yielded a *p*-value of 0.073, indicating good calibration. The decision curve demonstrated that, within the threshold probability range of 6 to 100%, the net benefit of using this model to guide intervention was higher than that of the “universal intervention” and “no intervene” strategies (Figure 6).

### 3.6 SHAP analysis results

**Figure 7:**
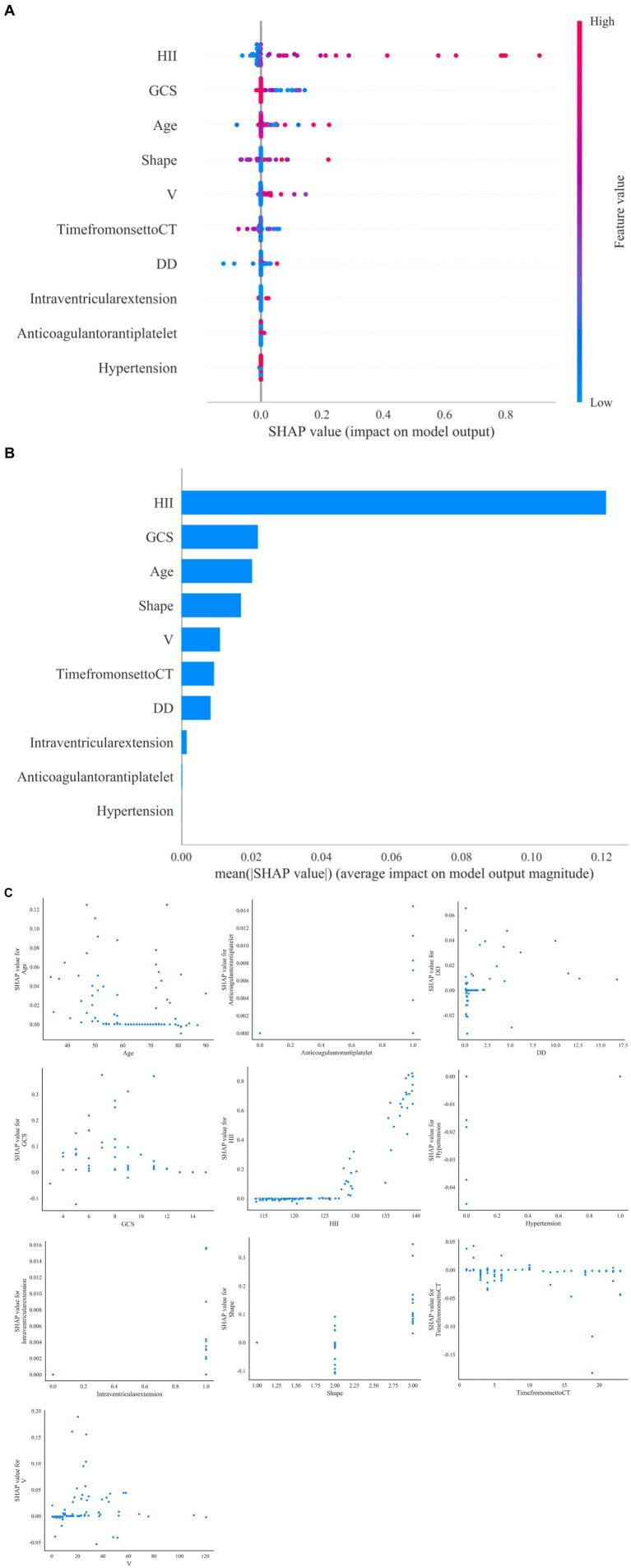
**(A)** SHAP summary plot of the 10-variable GBDT model. **(B)** SHAP importance plot of the 10-variable GBDT model. **(C)** SHAP scatter plot of the 10-variable GBDT model.

The SHAP summary plot and SHAP importance plot showed that the top three variables with the greatest influence on the model’s output were, in order, HII, GCS score, and age, followed by hematoma shape, hematoma volume, time from onset to CT scan, D-dimer, intraventricular hemorrhage, history of anticoagulant use, and history of hypertension. SHAP dependency plot analysis revealed the following: (1) Higher HII values correlate with higher SHAP values, indicating that a high HII was a risk factor for rebleeding; (2) Lower GCS scores correlate with higher SHAP values, suggesting that more severe neurological impairment was associated with a higher risk of rebleeding; (3) Irregular hematoma morphology (increased Shape value) corresponded to higher SHAP values; (4) The shorter the time from onset to CT scan, the higher the SHAP score; (5) The larger the hematoma volume, the higher the SHAP score; (6) The effect of age on the SHAP score was nonlinear, with relatively higher SHAP scores observed in middle age (approximately 40–60 years) (Figure 7).

### 3.7 Prediction website

**Figure 8:**
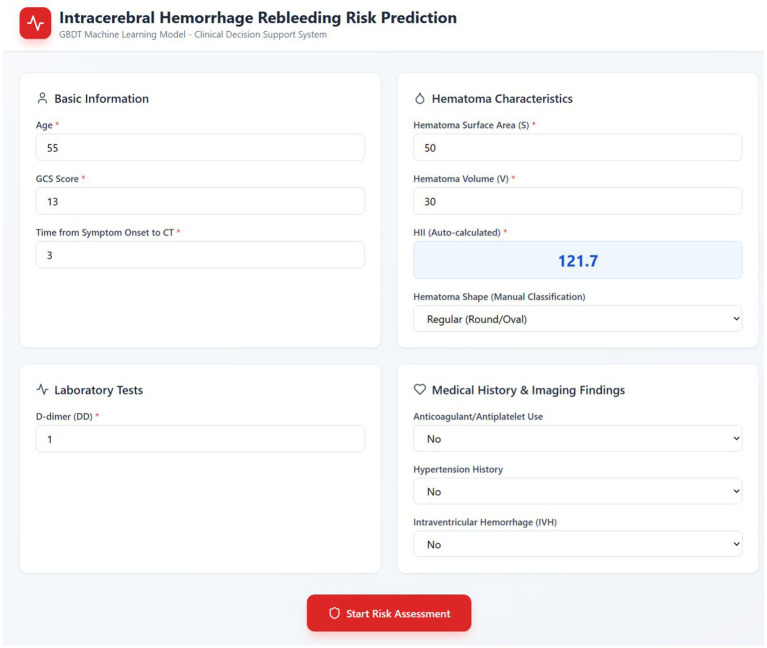
User interface of the online HII-based prediction tool for acute-phase rebleeding in intracerebral hemorrhage.

A web-based tool for predicting the risk of rebleeding in intracerebral hemorrhage was developed in this study, accessible at: https://uuqbqxk49i38.space.minimaxi.com. The tool requires no software or plug-in installation; clinicians can simply access it via a web browser, enter data, and obtain real-time prediction results. With its low operational threshold, it is suitable for various clinical settings, including emergency departments and neurology wards, and holds significant potential for widespread application (This tool is intended solely for clinical decision support and should not replace professional medical judgment. All treatment decisions should be based on a comprehensive clinical assessment by qualified healthcare professionals. This model was developed using single-center retrospective data, and external validation is currently ongoing.) (Figure 8).

## 4 Discussion

### 4.1 Research findings

In this study, systematic univariate analysis, Lasso regression, and multi-model comparisons confirmed that the GBDT algorithm demonstrated superior performance in predicting the risk of rebleeding during the acute phase of intracerebral hemorrhage. Ultimately, a GBDT predictive model comprising 10 clinically accessible indicators was developed. Bootstrap internal validation showed that the model’s AUC reached 0.964 (95% CI 0.939 ~ 0.982), indicating that the model possesses good stability and generalization ability. It can effectively distinguish between high-risk and low-risk patients, thereby providing decision support for early clinical intervention. The main innovations of this study are as follows: (1) A systematic comparison of the performance of nine mainstream machine learning algorithms in predicting HE, confirming that GBDT outperforms the other algorithms; (2) The introduction of the SHAP interpretability framework, which makes complex “black box” models transparent and interpretable, thereby enhancing clinicians’ understanding of and confidence in the prediction results; (3) The use of bootstrap resampling validation to ensure the model’s robustness; and (4) The development of a predictive website.

### 4.2 Risk factor analysis

Six core predictive variables were identified through Lasso regression in this study, and their order of importance was further validated via SHAP analysis. HII (hematoma heterogeneity index), GCS score, and age ranked in the top three, which is generally consistent with the results of previous studies. HII was the most important predictor in this study. Calculated using 3D Slicer software, it is defined as the ratio of the hematoma surface area to the square root of its volume (S/V ratio) and reflects the degree of irregularity and internal heterogeneity of the hematoma (20). In this study, a semi-automated segmentation method was employed, based on the density difference between hematoma CT values (30 ~ 80 HU) and normal brain parenchyma. Partial volume effects were manually corrected to ensure the accuracy of HII calculations. Previous studies have shown that hematoma heterogeneity was closely associated with active bleeding and hematoma instability (27, 28). Barras et al. found that patients with high HII values had a significantly increased risk of hematoma expansion. The results of this study further validated this finding; the SHAP-based plot showed a positive correlation between HII values and the risk of rebleeding, suggesting that clinicians should closely monitor patients with heterogeneous hematoma density and multiple low-density areas on CT scans. The GCS score was a classic indicator of neurological function. This study found that a lower GCS score (indicating more severe neurological impairment) was associated with a higher risk of rebleeding. This is consistent with previous studies, as patients with low GCS scores often indicate massive hemorrhage, deep-seated lesions, or the presence of cerebral herniation, which naturally increases the risk of hematoma expansion (5) Furthermore, the GCS score can serve as a comprehensive indicator of a patient’s overall clinical severity and is intrinsically linked to rebleeding. An irregular hematoma morphology (lobed or multifocal) was a recognized radiological predictor of hematoma expansion. In this study, the definition of irregular hematoma morphology was based on previous literature (29), with regular morphology (circular or oval) defined as Shape = 1, irregular morphology (lobed or irregular) defined as Shape = 2, and mixed density or island sign defined as Shape = 3. The SHAP dependency plot showed that irregular shapes correspond to higher SHAP values, suggesting an increased risk of rebleeding. This aligns with the pathophysiological mechanisms reflected by hematoma morphology: irregular shapes often indicate rupture of multiple blood vessels or the presence of multiple bleeding foci, making rebleeding more likely. Time from onset to CT scan is another important predictive factor. This study found that the shorter the time from onset to CT, the higher the SHAP score, suggesting that patients presenting early actually have a higher risk of rebleeding. This seemingly contradictory result may reflect two factors: first, patients who seek medical attention rapidly after onset often have more severe and rapidly progressing conditions and are inherently part of a high-risk population; second, early CT may capture the “early stage” before hematoma expansion, rather than the true baseline state (30). Therefore, for patients presenting early after onset, clinicians should be particularly vigilant regarding the risk of rebleeding and, if necessary, repeat imaging within 24 h. Hematoma volume is an important prognostic factor for intracerebral hemorrhage; multiple previous studies have confirmed a positive correlation between baseline hematoma volume and hematoma expansion (31) The results of this study are consistent with this finding: the larger the hematoma volume, the higher the SHAP score, and the greater the risk of rebleeding. This may be related to the mechanical compression effect of large hematomas on surrounding blood vessels and the poor stability of the hematoma itself. The nonlinear effect of age is a unique finding of this study. The SHAP dependency plot showed that SHAP values are relatively high in middle age (approximately 40 ~ 60 years), whereas they decrease in elderly patients (>70 years). This nonlinear feature aligns with the modeling philosophy of Asteris et al. (32), where complex disease outcomes were considered a multifactorial interactive surface, not merely a linear combination. This phenomenon may be related to the pathophysiological characteristics of patients in different age groups: middle-aged patients may have an increased risk due to a longer duration of vascular risk factors such as hypertension and severe damage to the vascular wall; whereas in elderly patients, the relationship between age and rebleeding is more complex due to the presence of multiple competing factors, such as an increased incidence of cerebral vascular amyloidosis and coagulation abnormalities (2). Notably, the model in this study was constructed using admission baseline data to predict the risk of early rebleeding in patients upon admission and before surgical intervention. All variables incorporated into the model were baseline indicators obtained at admission. Subsequent surgical decisions were made after risk prediction, which would neither affect the distribution of baseline data nor interfere with the prediction of early rebleeding risk.

### 4.3 Model performance evaluation

The 10-variable GBDT model developed in this study achieved an AUC of 0.85 and an accuracy of 0.8829 on the independent test set, significantly outperforming the traditional logistic regression model. Asteris et al. (33) used only five genetic variants plus age and gender, a total of seven features, in 133 COVID-19 patients and achieved an ICU outcome prediction accuracy of 89.47% with an artificial neural network, suggesting that a stable model can be built with a moderate sample size when the input features are strongly associated with the outcome. As an ensemble learning method, GBDT can automatically capture nonlinear relationships and interaction effects among variables, which is the primary reason for its superior predictive performance compared to logistic regression (13). For example, GBDT may automatically identify that the combination of “high HII and low GCS” significantly increases the risk of rebleeding, whereas in traditional logistic regression, such an interaction effect requires the manual addition of an interaction term to be captured. The results of the internal Bootstrap validation showed that the mean AUC reached 0.964, with a 95% confidence interval of 0.939 to 0.982, indicating that the model exhibits good stability. It is worth noting that there is a certain discrepancy between the AUC of the test set (0.8965) and the AUC of the Bootstrap validation (0.964). This may be related to sampling variance caused by the small sample size of the test set (*n* = 110); Moreover, Bootstrap internal validation resamples from the training set with overlapping samples, tending to overestimate model performance; an independent test set, entirely separate from the training data, reflects generalization ability more objectively. However, overall, the model’s performance remains at a high level. The calibration curve analysis showed good agreement between the model’s predicted probabilities and the actual incidence rates (Hosmer-Lemeshow *p* = 0.073), indicating that the model not only has good discriminatory power but also exhibits satisfactory calibration performance. Decision curve analysis further indicated that, within a threshold probability range of 6 to 100%, using this model to guide clinical decision-making yields a net benefit, demonstrating potential clinical utility. Most previous logistic regression-based HE prediction models have had AUC values ranging from 0.70 to 0.85 (36, 37), whereas the GBDT model in this study showed significantly better performance. The BAT score is a widely recognized scoring system for predicting HE, with an AUC of approximately 0.80 (10). The model performance in this study outperformed the BAT score, suggesting that machine learning algorithms have an advantage in predicting HE. Nevertheless, it should be noted that most previous studies used hematoma expansion of ≥33% as the definition criterion, whereas this study adopted a stricter criterion (≥33% or an increase of ≥6 mL); this may have led to differences in the study populations, and caution is warranted when directly comparing AUC values across studies. Furthermore, the introduction of SHAP for model interpretation in this study addresses the clinical application barrier posed by the “black box” problem of machine learning models.

### 4.4 Limitations

This study has certain limitations: (1) It is a single-center, retrospective study, which may be subject to selection bias and confounding factors; (2) The sample size is relatively small (*n* = 368); although Bootstrap validation was performed, further confirmation using external, multicenter data is still needed; (3) Some potential confounding factors were not included in the analysis, such as the specific types and doses of anticoagulants, blood pressure control levels, and bleeding sites; (4) This study constitutes an internal validation and lacks an external validation dataset. Furthermore, the HII calculation method used in this study requires specialized image analysis software, and its clinical accessibility needs to be improved. Future research could explore automated HII calculation methods based on radiomics to further enhance the model’s clinical applicability.(5) In addition to the limitations related to study design, several limitations inherent to machine learning methodologies should also be acknowledged. First, ML models are highly data-dependent; this may lead to class imbalance, and extreme values of certain parameters may be underrepresented. Second, although Bootstrap validation confirmed the internal stability of the model, the sample overlap introduced by resampling may overestimate performance, and the lack of external validation limits generalizability. Third, despite the use of SHAP to mitigate the “black box” problem, the decision logic of GBDT remains complex, and SHAP values reflect associative rather than causal relationships.

### 4.5 Clinical implications and future directions

The GBDT predictive model developed in this study has the potential to serve as a clinical decision-support tool, helping physicians identify high-risk patients at an early stage and formulate personalized monitoring and intervention strategies. For example, for patients predicted by the model to be at high risk, closer imaging monitoring, stricter blood pressure control, or early use of hemostatic agents may be considered. Future research could focus on the following areas: (1) conducting prospective multicenter studies to further validate the model’s performance; (2) incorporating additional imaging features (such as CT texture analysis and CTA signs) to improve predictive accuracy; (3) exploring automated feature extraction methods based on deep learning; (4) 3D-Slicer is free and open-source software, but its promotion in primary hospitals is limited by a lack of trained operators, time-consuming image post-processing, and constrained hardware conditions. We propose to develop a simplified HII calculation workflow in the future, integrate it into routine imaging systems, and thereby lower the threshold for its use at the primary care level. We also plan to develop a portable risk-assessment application to facilitate use at the clinical frontline; (5) By drawing on ensemble refinement algorithms such as DERGA (33), a small set of core indicators with the highest predictive value can be selected from the existing 35 ~ 40 multi-domain variables; for instance, Asteris et al. achieved 97% accuracy in predicting COVID-19 outcomes using only four routine blood parameters (34), thereby enabling the construction of a more concise bedside tool. In addition, feature importance ranking methods such as the *α*-index (35) can be introduced to achieve automatic dimensionality reduction and model optimization for high-dimensional variables.

## 5 Conclusion

The GBDT model developed in this study, based on 10 clinical indicators, can effectively predict the risk of rebleeding during the acute phase of intracerebral hemorrhage. Model robustness was confirmed by internal Bootstrap validation, and SHAP analysis provided a clear explanation of variable contributions. This model has the potential to serve as a clinical decision-support tool, helping clinicians identify high-risk patients early and develop personalized monitoring and intervention strategies.

## Data Availability

The original contributions presented in the study are included in the article/supplementary material, further inquiries can be directed to the corresponding author.
